# The Proprotein Convertase KPC-1/Furin Controls Branching and Self-avoidance of Sensory Dendrites in *Caenorhabditis elegans*

**DOI:** 10.1371/journal.pgen.1004657

**Published:** 2014-09-18

**Authors:** Yehuda Salzberg, Nelson J. Ramirez-Suarez, Hannes E. Bülow

**Affiliations:** 1 Department of Genetics, Albert Einstein College of Medicine of Yeshiva University, Bronx, New York, United States of America; 2 Dominick P. Purpura Department of Neuroscience, Albert Einstein College of Medicine of Yeshiva University, Bronx, New York, United States of America; University of California San Diego, United States of America

## Abstract

Animals sample their environment through sensory neurons with often elaborately branched endings named dendritic arbors. In a genetic screen for genes involved in the development of the highly arborized somatosensory PVD neuron in *C. elegans*, we have identified mutations in *kpc-1*, which encodes the homolog of the proprotein convertase furin. We show that *kpc*-*1*/furin is necessary to promote the formation of higher order dendritic branches in PVD and to ensure self-avoidance of sister branches, but is likely not required during maintenance of dendritic arbors. A reporter for *kpc-1*/furin is expressed in neurons (including PVD) and *kpc*-*1*/furin can function cell-autonomously in PVD neurons to control patterning of dendritic arbors. Moreover, we show that *kpc*-*1*/furin also regulates the development of other neurons in all major neuronal classes in *C. elegans*, including aspects of branching and extension of neurites as well as cell positioning. Our data suggest that these developmental functions require proteolytic activity of KPC-1/furin. Recently, the skin-derived MNR-1/menorin and the neural cell adhesion molecule SAX-7/L1CAM have been shown to act as a tripartite complex with the leucine rich transmembrane receptor DMA-1 on PVD mechanosensory to orchestrate the patterning of dendritic branches. Genetic analyses show that *kpc-1*/furin functions in a pathway with MNR-1/menorin, SAX-7/L1CAM and DMA-1 to control dendritic branch formation and extension of PVD neurons. We propose that KPC-1/furin acts in concert with the ‘menorin’ pathway to control branching and growth of somatosensory dendrites in PVD.

## Author Summary

Sensory neurons receive input from other neurons or sample their environment through elaborate structures termed dendritic trees. The correct patterning of dendritic trees is crucial for the proper function of the nervous system, and ample evidence points to the involvement of dendritic defects in a wide range of neuropsychiatric diseases. However, we still do not understand fully how this process is regulated at the molecular level. We discovered an important role for the protein-processing enzyme KPC-1/furin in the development of touch-sensitive dendritic trees in the roundworm *C. elegans*. Animals lacking this enzyme show multiple defects in the size, shape and number of these dendritic branches as well as other neurons. We further show that the gene encoding KPC-1 is expressed widely in the nervous system and that it is required within the branching neuron to exert its function on dendritic growth. Finally, we reveal a genetic connection between KPC-1 function and genes of the menorin pathway, which was recently discovered to also play an essential role in dendrite development. Thus, our findings add new insight into the molecular understanding of dendrite formation.

## Introduction

Multicellular organisms sense their environment through specialized nerve cells termed sensory neurons. The diversity in shape and structure that dendrites of sensory neurons (hereafter named ‘dendritic arbors’) assume reflects the variety of stimuli they receive [1]. Work over the past two decades has established that dendritic arbor development of sensory neurons relies on conserved molecular mechanisms [2], [3]. However, dendritic arbors do not only exist in the periphery, but also in the central nervous system where they function to receive synaptic input from other neurons. The molecules and mechanisms that orchestrate dendritic arbor development, e.g. cell adhesion molecules and molecular motor proteins, are often similar between the peripheral and central nervous system [see ref. 3 for these and more examples]. Importantly, failed dendrite development has been linked to neurological diseases of the central nervous system, ranging from Autism Spectrum Disorders (ASD) to Schizophrenia [4], [5].

**Figure 1:**
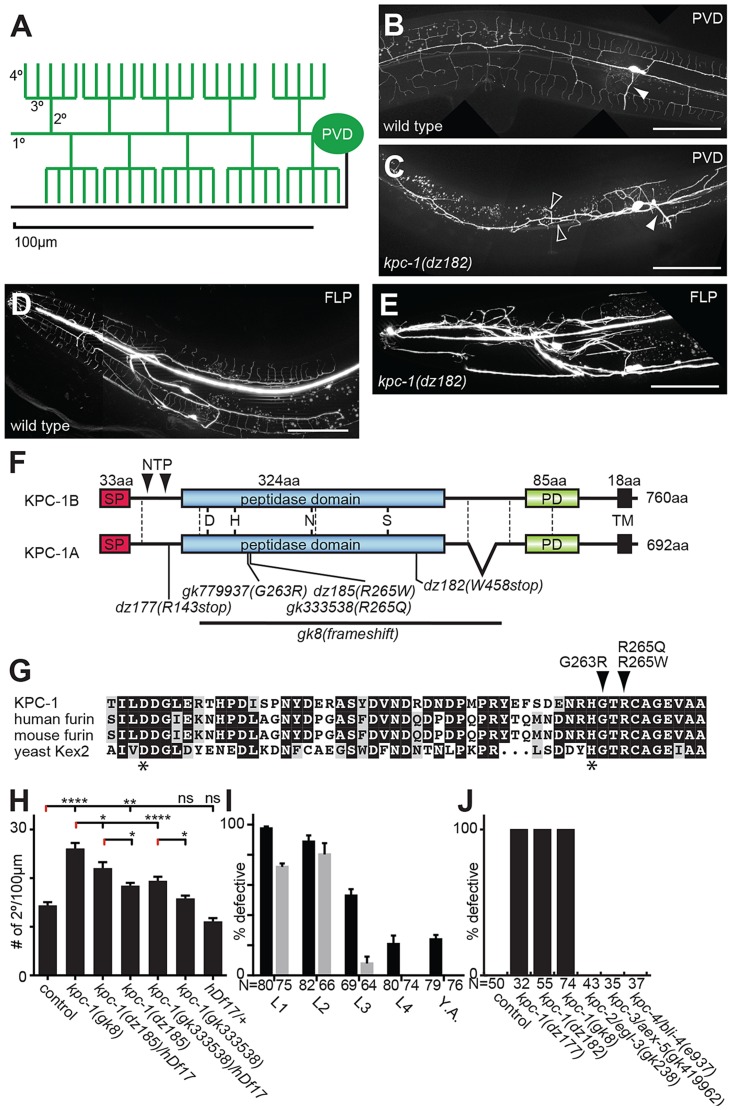
KPC-1 is required for development of dendritic arbors. **A** Schematic of the PVD somatosensory neuron with dendrites (including primary/1°, secondary/2°, tertiary/3° and quaternary/4° branches) and the axon indicated in green and black, respectively. **B–C** Lateral view of an adult wild type (B) and *kpc-1(dz182)* mutant animal (C). PVD sensory neurons are visualized by a fluorescent reporter (*wdIs52*). Anterior is to the left and scale bars indicate 50 µm in all panels. An arrowhead denotes the PVD axon. Open arrow heads indicate ectopic 3° branches. **D–E** Images of an adult wild type animal (D) and a *kpc-1(dz182)* mutant animal (E). FLP sensory neurons are visualized by a fluorescent reporter (*muIs32*). **F** Schematic of KPC-1 with domains (SP: signal peptide, PD: P domain, TM: transmembrane domain) and exon boundaries (dashed lines) indicated. KPC-1 exists in a long and a short splice variant. All experiments were conducted with the short KPC-1A variant. Mutant alleles and predicted molecular changes are shown. NTP (N terminal propeptide) indicates cleavage sites that are necessary for autoproteolytic activation of the proprotein. **G** Partial sequence alignment of the peptidase domain in KPC-1 (NP_001021101), human furin (NP_002560.1), mouse furin (NP_001074923.1), and yeast Kex2 (NP_014161) protein. Asterisks indicate the aspartic acid and histidine of the catalytic triad. Alignments were obtained using Multalin (http://multalin.toulouse.inra.fr) and rendered with Boxshade (http://www.ch.embnet.org). For a Full alignment see Figure S1. **H** Quantification of the number of secondary branches in the genotypes indicated. *hDf17* is a chromosome I deficiency allele that deletes the entire *kpc-1* locus. Data are represented as mean +/− SEM. Statistical comparisons were performed using one-sided ANOVA with the Tukey correction. Statistical significance is indicated (*, *P*≤0.05; **, *P*≤0.01; ****, *P*≤0.0001, ns: not significant (*P*>0.05)). N = 22 animals (302 dendritic branches) of wild type control; N = 22 animals (548 dendritic branches) of *kpc-1(gk8)*; N = 16 animals (331 dendritic branches) of *kpc-1(dz185)/hDf17*; N = 26 animals (461 dendritic branches) of *kpc-1(dz185)*, N = 16 animals (292 dendritic branches) of *kpc-1(gk333538)/hDf17*, N = 26 animals (396 dendritic branches) of *kpc-1(gk333538)*. **I** Quantification of animals with defects in PVD at the young adult stage following RNAi mediated knock down of *kpc-1* (grey bars) or GFP as a control for RNAi efficacy (black bars) using the hypersensitive *eri-1(mg366); wdIs52* reporter background at 15C°. All animals were assayed as 2 day old adults and the developmental stage at which knock down was initiated is shown. Animals were arbitrarily defined as defective, if they displayed excessive ectopic branching proximal to the cell body, coupled with complete lack of quaternary branches anterior to the vulva or, GFP was absent in PVD. Note, that while knock down of GFP initiated at late developmental stages is still effective, no effects are seen at these stages as a result of *kpc-1* knock down. **J** Quantification of animals with defects in PVD in the genotypes indicated (See also Figure S6). Defective animals were defined as in I.

The understanding of sensory dendrite morphogenesis has been greatly advanced through studies in the fly *Drosophila melanogaster* (reviewed in [3]). These studies revealed the importance of transcriptional cascades, cytoskeletal proteins, the secretory pathway, microtubular transport, and the basement membrane for development of the so-called dendritic arborization (da) neurons in flies [3], [6]–[8]. In the nematode *Caenorhabditis elegans*, the PVD mechanosensory neurons have been used to study development of dendritic arbors owing to their stereotypic branching patterns (Figure 1A,B) [9], [10]. A PVD cell on each side of the animal sends a primary dendrite both anteriorly and posteriorly that branches off perpendicular higher order branches in a stereotypical fashion (Figure 1A, B). The resulting structures resemble candelabra and have hence been named menorahs [11]. Like fly da neurons, transcriptional cascades and microtubule based motor proteins have been shown to play a role during PVD dendritic arbor formation suggesting that basic principles of dendritic arbor formation are evolutionarily conserved [12]–[14]. Recently, a tripartite complex consisting of the extracellular molecule MNR-1/menorin, the neural cell adhesion molecule SAX-7/L1CAM both of which act from the hypodermis (skin) and the leucine rich repeat DMA-1/LRR transmembrane receptor on PVD dendrites have been shown to be critical for PVD development [15], [16].

Here we report the identification of the furin homolog *kpc-1* in *C. elegans* as a factor that acts in concert with the ‘menorin’ pathway to shape sensory dendrite development. Furin is a serine protease of the proprotein convertase family, that following autocatalytic activation cleaves proteins at characteristic dibasic motifs (reviewed in [17]–[19]). We show that in *C. elegans* a reporter for *kpc-1*/furin is expressed broadly in the nervous system and is necessary for multiple neuronal positioning, neurite extension and branching events, including PVD dendritic growth and self-avoidance. Moreover, *kpc-1* can act cell autonomously to shape PVD dendrites in a manner that is dependent on the ‘menorin’ pathway.

## Results

In a screen for genes involved in PVD development [16] we also identified mutant alleles of the proprotein convertase *kpc-1*, which encodes the *C. elegans* furin homolog based on sequence similarity and domain organization [20], [21]. Mutant animals show no obvious morphological or defects in body size. However, they do display severely defective dendritic arbors in both PVD and the analogous anterior FLP neurons (Figure 1B–E). The identified recessive alleles include two nonsense (*dz177*, *dz182*) and a missense allele (*dz185*) (Figure 1F, Figure S1). We also obtained a deletion (*gk8*) and two additional missense alleles (*gk333538*, *gk779937*) (Figure 1F,G, Figure S1). The deletion and nonsense alleles truncate or delete the catalytic domain of KPC-1/furin, respectively, and their phenotypes seem indistinguishable in severity, suggesting that all three alleles represent strong if not complete loss of function alleles. The missense alleles *dz185* and *gk333538* are hypomorphic alleles because their phenotype was (1) less severe when compared to the presumptive *gk8* null allele and (2) more severe when placed in trans to a deficiency (*hdf17*) that spans the genomic region of *kpc-1*/furin (Figure 1H). The missense mutations affect residues in a perfectly conserved alpha helix that positions the histidine of the catalytic triad in furin [22] (Figure 1G), suggesting that proteolytic activity of KPC-1/furin is necessary for PVD development.

To define when *kpc-1*/furin function is required during development we first conducted timed RNAi experiments as described [16]. We found that knock down of *kpc-1*/furin starting at early larval stages when PVD sensory dendrites begin to develop [13] resulted in robust defects in PVD in adult animals (Figure 1I). In contrast, knock down starting at the L3 stage resulted in weaker phenotypes in adults (Figure 1I). RNAi mediated knockdown initiated only 12–24 h later at the L4 larval stage failed to result in any PVD defects. Importantly, RNAi mediated knockdown of GFP in PVD neurons suggested that changes in RNAi efficacy cannot account for lack of defects upon induction of *kpc-1* knock down at later larval stages (Figure 1I). Consistent with these observations we find defects in PVD dendrite arborization in *kpc-1* mutant animals already during the L3 larval stage rather than later as a result of a maintenance defect (Figure S2). Collectively, these findings suggest that *kpc-1*/furin functions during the earlier stages of PVD development and may not play a major role in dendrite maintenance.

**Figure 2:**
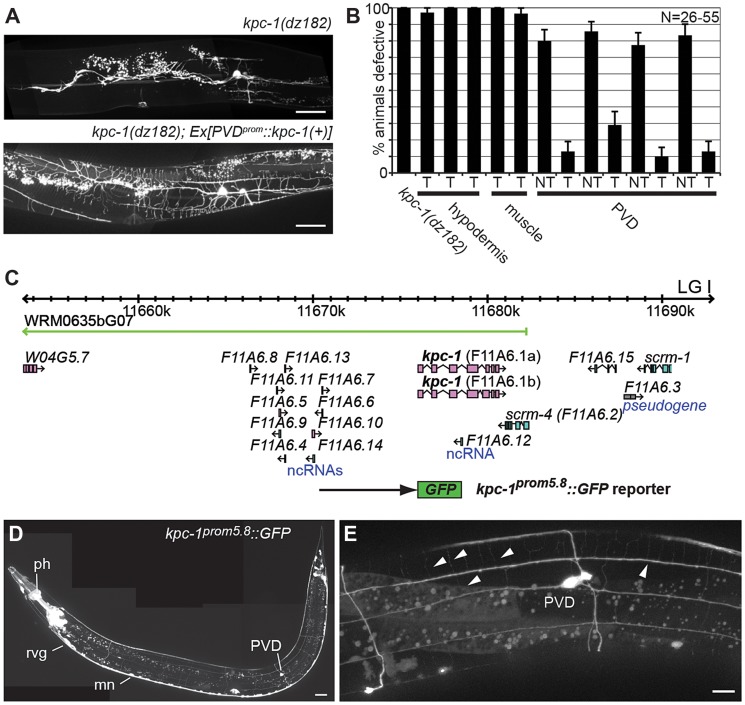
KPC-1 functions in PVD to shape dendritic arbors. **A** Epifluorescence micrographs of *kpc-1(dz182)* mutant animals and transgenically rescued *kpc-1(dz182)* animals. Scale bars indicate 50 µm. **B** Quantification of transgenic rescue. Transgenic (T) and nontransgenic (NT) siblings were quantified in *kpc-1* mutant animals in which a *kpc-1* wild type DNA was driven from a hypodermal (*dpy-7^prom^*) [31], muscle (*myo-3^prom^*) [32] or PVD specific promoter (*ser-2^prom3^*) [9]. Defective animals were defined as in Figure 1I. **C** Schematic of the *kpc-1* locus on linkage group (LG) I. The extent of the rescuing fosmid WRM0635bG07 is indicated in green as is the transcriptional *kpc-1::GFP* reporter that contains 5.8 kb of regulatory sequence upstream of the ATG (*kpc-1^prom5.8^::GFP*). A transcriptional reporter containing 2.8 kb of regulatory sequence (*kpc-1^prom2.8^::GFP*) was not expressed in PVD suggesting that regulatory elements for PVD expression reside between −2.8 kb and −5.8 kb (Figure S4). **D** Composite epifluorescent micrograph of an adult transgenic animal carrying the *kpc-1^prom5.8^::GFP* transgene. Expression is seen in the pharynx (ph) and the nervous system, *e.g.* the retrovesicular ganglion (rvg), ventral cord motor neurons (mn) and in PVD mechanosensory neurons. In addition, expression is seen in vulval epithelial tissues as well as the spermatheca (not shown here). A scale bar indicates 30 µm. **E** Higher magnification epifluorescent micrograph of an adult transgenic animal carrying the *kpc-1^prom5.8^::GFP* transgene. Expression is seen in PVD and its dendritic tree (arrowheads). A scale bar indicates 10 µm.

We next sought to determine whether *kpc-1*/furin functions in PVD neurons or in surrounding tissues such as the hypodermis (skin) or muscle. To this end we first conducted transgenic rescue experiments. We found rescue of the PVD defects in *kpc-1* mutants when a *kpc-1* cDNA was expressed under control of a PVD-specific heterologous promoter but not when expressed in muscle or the hypodermis (Figure 2A,B). Expression of this PVD-specific *kpc-1* transgene in a wild-type background did not have any detectable effect on PVD architecture (Figure S3). To further investigate where *kpc-1* may function we constructed transgenic animals that carried a *kpc-1* reporter where a 5.8 Kb fragment of the endogenous upstream regulatory region of *kpc-1* drives expression of green fluorescent protein. This transcriptional *kpc-1^prom5.8^::GFP* reporter was widely expressed from early developmental stages in the nervous system including in PVD (Figure 2C–E, Figure S4). We conclude that *kpc-1*/furin acts cell-autonomously to shape the dendritic arbors of PVD neurons.

**Figure 3:**
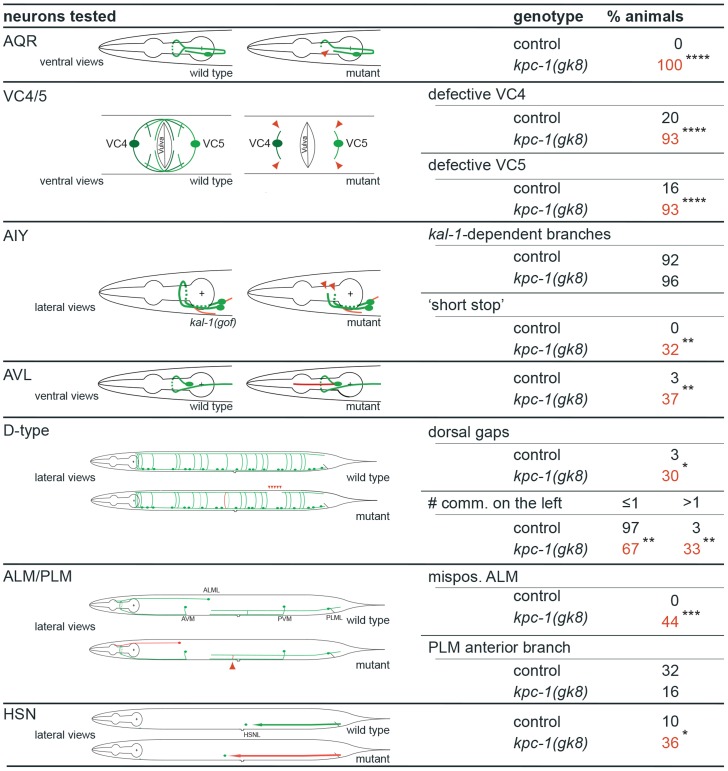
*kpc-1* displays defects in neurite branching, extension and cell positioning of certain sensory neurons, interneurons and motoneurons. Summary of neuronal defects observed in several classes of neurons. Schematics of normal neuronal morphology is shown and contrasted with abnormal neuronal morphology indicated in red or with red arrowheads. N = 30 in all cases except AIY and ALM (N = 25). Statistical significance was calculated using Fisher's exact test and is indicated (*, P≤*0.05*; **, P≤*0.01*; ***, P≤*0.001*; ****, P≤*0.0001*, ns: not significant (P>*0.05*)).

The observed extensive neuronal expression of the *kpc-1* reporter prompted us to examine other neuron types besides PVD for defects in migration, neurite extension and branching in *kpc-1* mutant animals. We detected significant defects in multiple neuron types in *kpc-1* mutants. For example, the sensory AQR neurons failed to branch appropriately in the nerve ring whereas VC motoneurons showed defects in the extension and formation of characteristic branches near the vulva (Figure 3). Yet, *kpc-1* did not appear to be required for all branching processes because branching in AIY interneurons as a result of overexpression of the secreted *kal-1* cell adhesion molecule [23] did not require *kpc-1* and, AVL neurons showed ectopic branches but no defects in normal branch formation (Figure 3). Whereas DA/DB motoneurons did not display obvious defects, D-type motoneurons showed gaps in the dorsal cord, possibly due to defective neurite extension, and increased numbers of inappropriately positioned commissures on the left side of the animals (Figure 3). Similarly, AIY interneurons displayed a characteristic axonal ‘short stop’ phenotype, likely as a result of defects in extension [23]. In addition, we observed neuronal cell positioning defects of ALM touch receptor neurons and HSN motoneurons (Figure 3). Thus, *kpc-1*'s function in nervous system development extends well beyond its role in dendrite morphogenesis and affects many but not all neurons in the major neuronal classes (sensory, motor and interneurons). Importantly, *kpc-1* appears to be involved in both control of neurite branch formation and extension (see also below).

**Figure 4:**
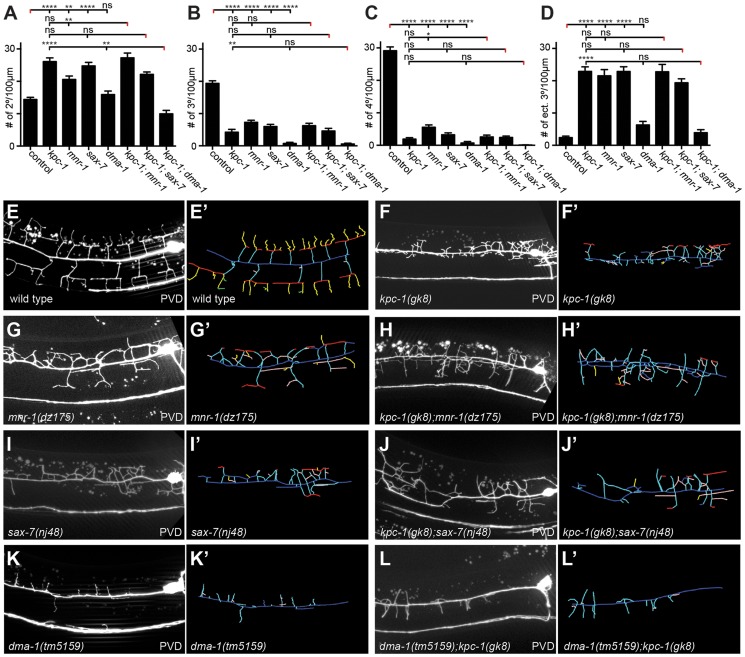
*kpc-1* acts in a genetic pathway with *mnr-1*/menorin, *sax-7*/L1CAM, and the leucine rich repeat (LRR) transmembrane receptor *dma-1*. **A–D** Quantification of branch numbers in *kpc-1(gk8)*, *mnr-1(dz175)*, *sax-7(nj48)*, and *dma-1(tm5159)* single and double mutant animals. Data are represented as mean +/− SEM of the number of branches found in the 100 µm segment immediately anterior to PVD cell body. Statistical comparisons were performed using one-sided ANOVA with the Tukey correction and statistical significance is indicated (*, *P*≤0.05; **, *P*≤0.01; ***, *P*≤0.001; ****, *P*≤0.0001, ns: not significant (*P*>0.05)). N = 21 animals (1371 dendritic branches) of wild type control; N = 21 animals (1158 dendritic branches) of *kpc-1(gk8)*; N = 21 animals (1154 dendritic branches) of *mnr-1(dz175)*; N = 22 animals (1251 dendritic branches) of *sax-7(nj48)*, N = 22 animals (520 dendritic branches) of *dma-1(tm5159)*, N = 19 animals (1119 dendritic branches) of *kpc-1(gk8)*; *mnr-1(dz175*), N = 25 animals (1213 dendritic branches) of *kpc-1(gk8)*; *sax-7(nj48)*, N = 22 animals (319 dendritic branches) of *kpc-1(gk8)*; *dma-1(tm5159)*. Data for wild type control and *kpc-1* are identical to Figure 1 and shown for comparison only. **E**–**L** maximum intensity projections of representative L3 larval animals of the genetic backgrounds as indicated, paired with schematics of the respective tracings (E′–L′). Traces were color coded as follows: dark blue: 1°, cyan: 2°, red: 3°, yellow: 4°, rose: ectopic 3°, dark blue: mispositioned 3° (combined with ectopic 3° for quantification purposes), green: 5°.

**Figure 5:**
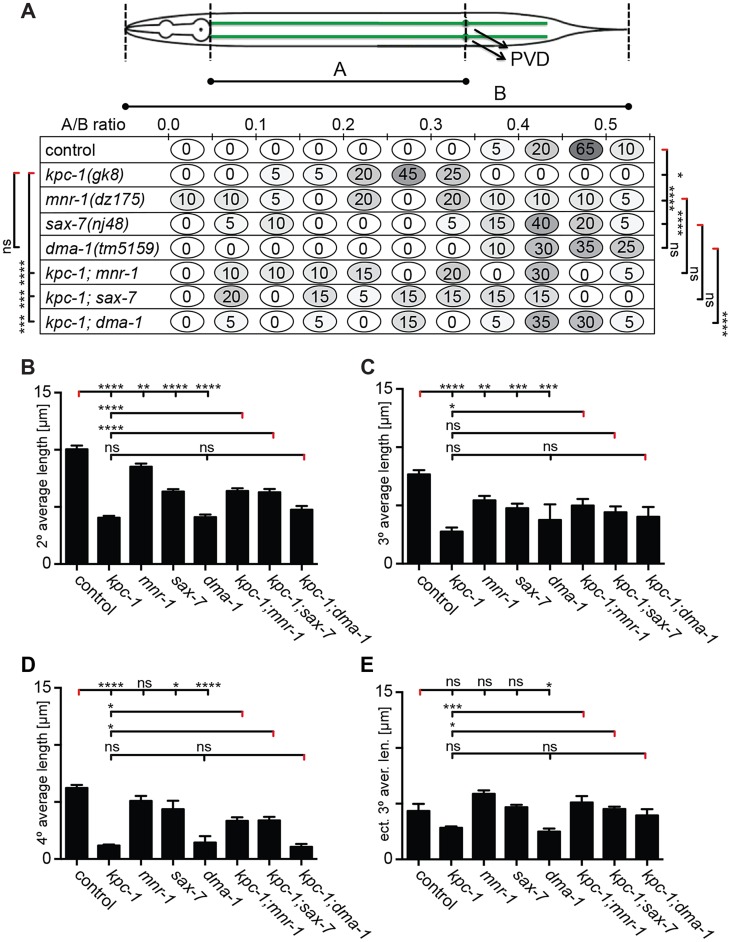
*kpc-1* controls dendrite branch extension. **A** Quantification of primary branch length, expressed as a ratio of A/B to normalize for animal size (as indicated in schematic) is shown for the genotypes indicated. Percentage of animals falling within a bin (of A/B ratio) is given (illustrated with the corresponding shade of grey). Statistical significance was calculated using the F test to compare variances (*, *P*≤0.05; **, *P*≤0.01; ***, *P*≤0.001; ****, *P*≤0.0001, ns: not significant (*P*>0.05). **B**–**E** Quantification of average branch length in *kpc-1(gk8)*, *mnr-1(dz175)*, *sax-7(nj48)*, and *dma-1(tm5159)* single and double mutant animals. Data are represented as mean +/− SEM. Statistical comparisons were performed using one-sided ANOVA with the Tukey correction. Statistical significance is indicated (*, *P*≤0.05; **, *P*≤0.01; ***, *P*≤0.001; ****, *P*≤0.0001, ns: not significant (*P*>0.05)). Number of animals and dendrites scored as in legend to Figure 4.

**Figure 6:**
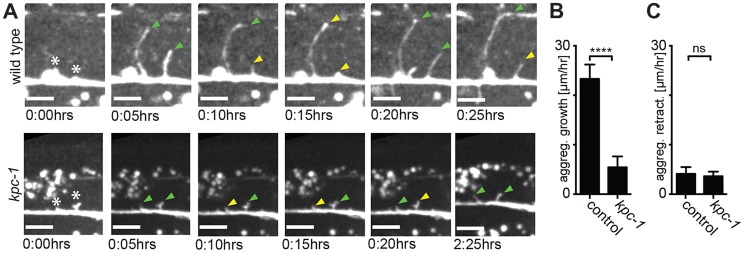
Dendrites in *kpc-1* display decreased speed of growth. **A** Time-lapse still images of a wild type (upper panel) and a *kpc-1(gk8)* mutant animal (lower panel), indicating the times after imaging was begun at the L3 stage. Asterisks mark the place of budding secondary dendrites being measured. Green arrowhead: secondary dendrites in active dendritic growth. Yellow arrowhead: secondary dendrites undergoing retraction. Note the limited growth of dendrites in *kpc-1* mutant animals even 2:25 hours after initiation. **B–C** Quantification of aggregate growth (B) and aggregate retraction speed (C) per hour in secondary branches from time lapse movies of the genotypes indicated. Data are represented as mean +/− SEM. Statistical comparisons were performed using the Student t-test and statistical significance indicated (***, *P*≤0.001; ns, not significant (*P*>0.05)).

To define the function of *kpc-1* in dendrite development in more detail we subjected mutants to a morphometric analysis of PVD dendrites. These studies showed an increase in secondary and ectopic tertiary branches with a concomitant decrease in the number of tertiary and quaternary branches in *kpc-1* mutants (Figure 4A–F). The secondary branches in *kpc-1* mutants often failed to reach the vicinity of the sublateral nerve cords (Figure 4F) where they normally bifurcate to form tertiary branches [13]. Instead, they frequently sprouted ectopic tertiary branches in places that normally do not support tertiary branches (Figure 4F). Interestingly, the average length of branches was significantly reduced in *kpc-1*/furin mutants (Figure 5A–E), as was the aggregate length of each branch order (2°, 3° and 4° branches) separately or combined (Figure S5). To distinguish whether this phenotype is a reflection of a decrease or increase in growth or retraction, we conducted time-lapse analyses of *kpc-1* mutants and measured growth and retraction of secondary branches. We found significantly slowed growth in *kpc-1* mutants compared to wild type animals whereas the speed of retraction remained unchanged (Figure 6, Movies S1 and S2). None of the observed PVD defects were shared in mutants of the three other proprotein convertases encoded in the *C. elegans* genome, including *kpc-2/egl-3*, *kpc-3/aex-5*, and *kpc-4/bli-4* [20] (Figure 1J, Figure S6), suggesting that these genes play individually no role during PVD development.

**Figure 7:**
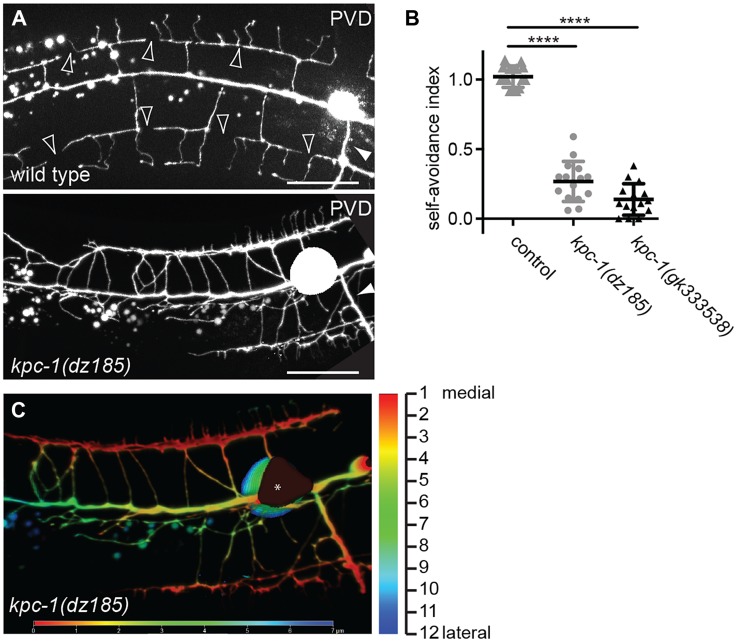
*kpc-1* serves a function in dendritic self-avoidance. **A** Epifluorescent micrographs of a lateral view of wild type and *kpc-1(dz185)* hypomorphic animals. Filled white arrowheads indicate axons and empty white arrowheads gaps between tertiary dendrites, which are known to be maintained by self-avoidance [33]. Scale bar is 20 µm. **B** Quantification of self-avoidance index which was determined as previously described [34] and is defined as the ratio of the number of gaps between adjacent tertiary branches divided by the number of candelabra in a 100 µm segment anterior to the cell body. Statistical comparisons were performed using the Student t-test and statistical significance is indicated (****, *P*≤0.0001). **C** Maximum intensity projection of a Z-stack (lateral view of a *kpc-1(dz815)* mutant shown in A) in which each optical section (total of 12 sections over 7.2 µm equaling 0.6 µm/section) was color-coded with warmer to colder colors for medial to lateral sections of the animal, respectively. Note that the overlapping tertiary dendrites appear in the same focal plane. Since the width of dendritic branches is approximately 200 nm [35], these data suggest that tertiary dendrites are closely apposed (within 0.6 µm) or directly touching. An asterisk marks an area of the cell body that is saturated due to maximum intensity projection.

In the hypomorphic alleles *kpc-1(gk333538)* and *kpc-1(dz185)* we found that tertiary and possibly other dendritic branches appeared to show substantial overlap and an apparent lack of self-avoidance (Figure 7A,B). These observations could indicate *bona fide* defects in self-avoidance of dendrites. Alternatively, mutant dendrites could be growing erroneously in three dimensions into hypodermal tissues, rather than growing within a confined two-dimensional plane and directly avoiding each other. Such observations have recently been made for drosophila dendrite arborization neurons whose growth failed to be restricted to a two-dimensional plane in integrin mutants but rather occurred in three dimensions [7], [8]. To distinguish between these two possibilities, we optically sectioned animals bearing a hypomorphic mutation in *kpc-1(dz185)* and coded each optical section with a different color. Thus, dendrite processes that share a focal plane display the same color whereas processes in different focal planes will display different colors. These analyses suggested that dendrites touch and overlap in the same focal planes in *kpc-1* hypomorphic mutant animals (Fig. 7C). Collectively, our data suggest that the normal function of *kpc-1* is to promote the formation of tertiary and quaternary branches and to maintain self-avoidance between dendrites. In addition, *kpc-1* functions in dendrite extension along the anterior-posterior and dorsal-ventral axes, likely by regulating the speed of growth.

**Figure 8:**
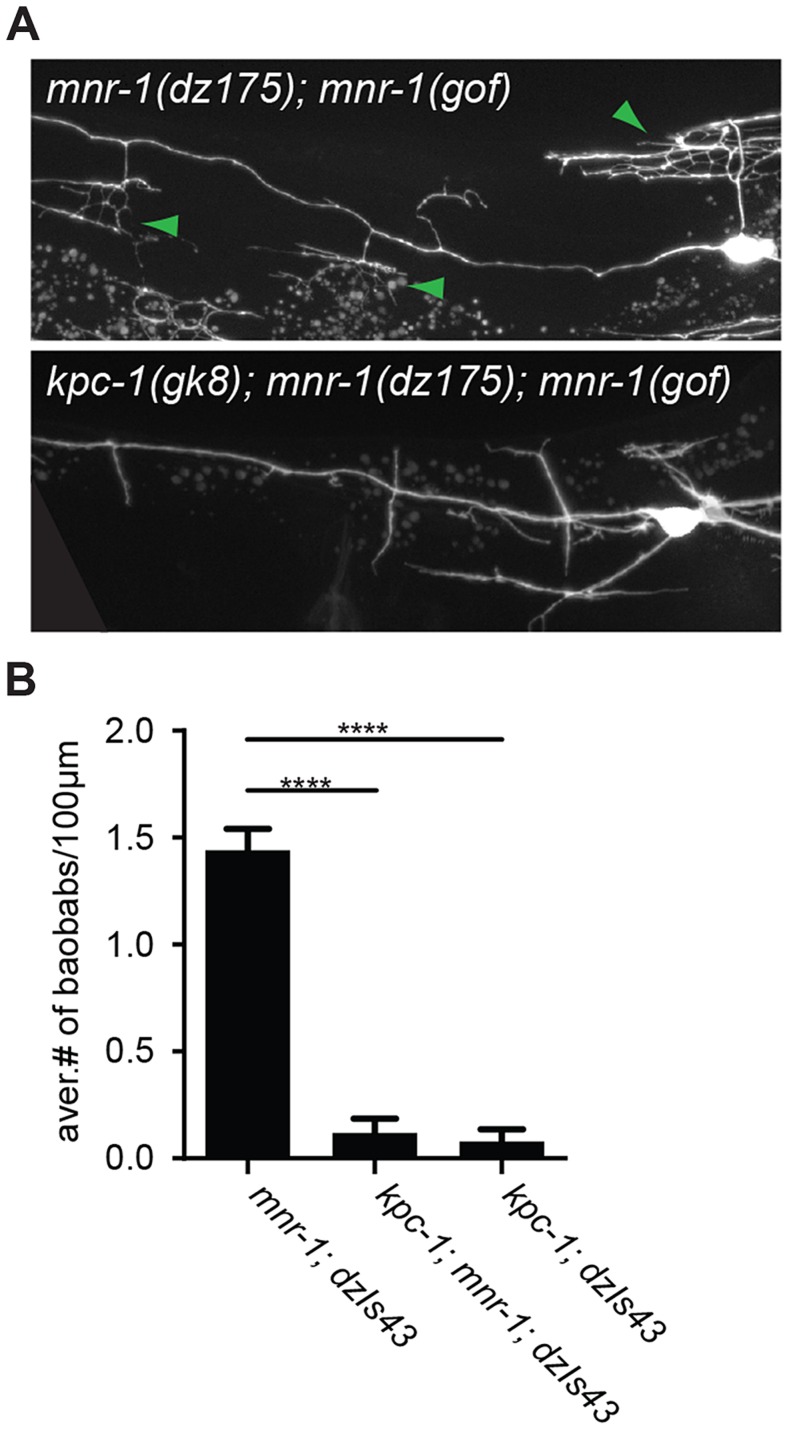
MNR-1/menorin requires *kpc-1* functions. **A** Epifluorescent micrographs of *mnr-1(dz175); mnr-1(gof)[dzIs43]* animals with characteristic ‘baobab’-like dendritic arbors (green arrowheads, upper panel) [16] that are absent upon loss of *kpc-1* (lower panel). **B** Quantification of ‘baobab’-like dendritic arbors. Mutant alleles used were *mnr-1(dz175)*, *dzIs43 (Is[myo-3^prom^::mnr-1])* and *kpc-1(gk8)*.

The dendrite phenotypes in *kpc-1* mutants were reminiscent of defects in mutants of the ‘menorin’ pathway, namely the cell surface molecule MNR-1/menorin, the neural cell adhesion molecule SAX-7/L1CAM and the leucine rich repeat containing transmembrane receptor DMA-1/LRR [15], [16]. We thus tested the genetic interactions between *kpc-1*/furin and each of these genes using the same morphometric criteria. Focusing first on the defects in branch formation in *kpc-1*/furin mutants (i.e. decreased tertiary and quaternary branching and increased secondary and ectopic tertiary branching) we found the *kpc-1* mutant phenotype not further enhanced in double mutants with either *mnr-1* or *sax-7*/L1CAM (Figure 4), suggesting that these genes act genetically in the same pathway. In contrast, *dma-1* appeared generally epistatic to *kpc-1* because the *kpc-1; dma-1* double mutant looked more similar to the *dma-1* single mutant (Figure 4). A possible exception was the reduced number of secondary branches in the *kpc-1; dma-1* double compared to both single mutants and to wild type animals (Figure 4A). This synthetic phenotype reveals possibly a cryptic or parallel function for *dma-1* in the formation of secondary branches that is only evident in the absence of *kpc-1*. To further investigate the genetic relationship between the menorin pathway and *kpc-1*, we used a previously described misexpression phenotype of *mnr-1*/menorin. When *mnr-1*/menorin is ectopically expressed in muscle, the resulting dendritic trees lack the characteristic menorah like morphology and, instead, display muscle-associated dendritic structures we named ‘baobabs’ because of their resemblance to baobab trees (Figure 8) [16]. We found that this *mnr-1*/menorin gain of function phenotype is fully dependent on *kpc-1* (Figure 8) as it is on *sax-7*/L1CAM and *dma-1*/LRR [16], demonstrating that *kpc-1* is required for the formation of *mnr-1*-dependent dendritic arbors. Taken together, these results suggest that for branch formation, *kpc-1*, *mnr-1/*menorin and *sax-7*/L1CAM act genetically in the same pathway and that *dma-1*/LRR is epistatic to *kpc-1* in a similar way as it is to *mnr-1/*menorin and *sax-7*/L1CAM [16].

With regard to branch length, *i.e.* branch extension, the situation is slightly different. The *kpc-1; dma-1* double mutant was indistinguishable from the *kpc-1* and *dma-1* single mutants, again demonstrating that both genes act genetically in the same pathway (Figure 5). In contrast, the reduced average length of higher-order branches in *kpc-1* mutants was partially suppressed in double mutants with *mnr-1*/menorin or sax*-7*/L1CAM but not with *dma-1*/LRR (Figure 5B–E). Similar observations were made for the primary branches. Primary branch length was measured from cell body to pharynx border and normalized relative to total body length to account for possible variability in body size (Figure 5A, top). The shorter primary branch length in *kpc-1* mutants was also partially suppressed by concomitant loss of *mnr-1*/menorin, *sax-7*/L1CAM, or *dma-1*/LRR (Figure 5A). Collectively, these results suggest that *kpc-1* acts genetically in the same and, possibly in part, a parallel pathway to *mnr-1*/menorin and *sax-7*/L1CAM to control dendrite patterning.

## Discussion

In a screen for genes required for patterning of the PVD somatosensory neuron, we isolated mutant alleles in *kpc-1*, which encodes the *C. elegans* furin homolog [20], [21]. The phenotypes in PVD patterning that we observed in *kpc-1*/furin mutants displayed striking similarities to mutants in the ‘menorin’ pathway, which comprises the three cell adhesion molecules MNR-1/menorin, SAX-7/L1CAM and DMA-1/LRR-TM [15], [16]. Our double mutant analyses revealed genetic interactions that were distinct for branch formation and extension. For branch formation, we suggest that *kpc-1*/furin acts in a genetic pathway with *mnr-1*/menorin, *sax-7*/L1CAM and *dma-1*/LRR. First, double mutants between *kpc-1*/furin and either *mnr-1*/menorin or *sax-7*/L1CAM were not more severe than the single mutants. Second, *dma-1*/LRR appeared generally more severe than the *kpc-1*/*mnr-1* or *kpc-1*/*sax-7* double mutants and epistatic to *kpc-1*/furin. Third, gain of function experiments demonstrated that *mnr-1*/menorin function requires *kpc-1*/furin. Overall, these genetic interactions are strikingly similar to those between *mnr-1*/menorin, *sax-7*/L1CAM and *dma-1*/LRR [15], [16] and suggest that *kpc-1*/furin, *mnr-1*/menorin and *sax-7*/L1CAM act in a linear pathway but that a parallel pathway may exist that also acts through *dma-1*/LRR during branch formation. For branch extension, the scenario is slightly different. First, *kpc-1*/furin mutant branch extension phenotypes were generally more similar to *dma-1*/LRR than to *mnr-1*/menorin or *sax-7*/L1CAM mutant phenotypes. Second, mutations in *mnr-1*/menorin or *sax-7*/L1CAM but not *dma-1*/LRR partially suppressed *kpc-1*/furin mutant branch extension phenotypes in higher order branches. This could suggest higher activity of a parallel pathway during extension that is normally inhibited by *mnr-1*/*sax-7*-function, but also requires *dma-1*/LRR. Taken together, the findings presented here suggest that *kpc-1* collaborates with the menorin pathway to sculpt the PVD dendritic arbor through distinct genetic mechanisms for branch formation and extension.

Our experiments showed that *kpc-1*/furin functions cell autonomously to coordinate formation, extension and self-avoidance of PVD somatosensory dendritic branches. However, the functions of *kpc-1*/furin are not limited to shaping PVD and FLP neurons during development. First, a recent report described a function for KPC-1 in the remodeling of sensory dendrites of IL2s (a set of sensory neurons in the head of *C. elegans*) as a result of changes in environmental conditions [21]. This report provided also evidence for a role of *kpc-1* in the patterning of PVD and FLP neurons. Second, our detailed survey of the neuroanatomy of several classes of neurons in *kpc-1*/furin mutants revealed that *kpc-1*/furin plays a more general role in nervous system development than previously acknowledged. For example, AIY interneurons display neurite extension defects and the D-type motoneurons exhibit gaps in the dorsal nerve cord that appear to be the result of defects in neurite extension. On the other hand, *kpc-1*/furin is required for the formation of certain characteristic neuronal branches like in AQR sensory neurons or VC4/5 motoneurons, while preventing ectopic branching in other neurons such as the AVL neuron. In addition, *kpc-1* functions during cell migration of the touch neuron ALM and HSN motoneurons. How could *kpc*-1/furin regulate such seemingly diverse developmental processes as formation and extension of neurite branches or neuronal positioning? One hint may come from our time-lapse analyses, which established that PVD branches grow slower in *kpc-1*/furin mutants compared to wild type animals. It remains unclear how KPC-1/Furin controls branch formation, extension or the speed of growth in such diverse cellular contexts on a mechanistic level. One possibility is that KPC-1 regulates extracellular adhesion of the neuron/growth cone to the substrate either directly or indirectly to mediate these functions, much like it has been suggested for metalloproteases (reviewed in [24]).

An important question is hence to determine the target(s) that are proteolytically processed by KPC-1/furin. In vertebrates, furin or furin-like proteases are known to cleave members of the TGFbeta family of morphogens as well as neuropeptides in the secretory pathway [17], [18]. Yet, mutations in genes required for neuropeptide processing and secretion, including the EGL-21/carboxypeptidase E, the PAMN-1/peptidyl-α-hydroxyglycine-α-amidating lyase [25] or *unc-31*/CAPS which is required for dense core vesicle secretion [26] did not result in comparable defects in PVD (Figure S6). Similarly, neither mutations in genes of the TGFbeta pathway (including the TGFbeta ligands *tig-2*, *dbl-1*, *unc-129* or the sole type II TGFbeta receptor *daf-4*) nor in the *C. elegans* homolog of the repulsive guidance molecule (RGM), known to be cleaved by furin [27] displayed similar defects in PVD (data not shown). Several of the genes that have been shown to be required for PVD development contain predicted cleavage sites for furin-like proprotein convertases (Table S3). Thus, a candidate gene approach testing those genes and alternative genetic or proteomic approaches will be required to identify the *in vivo* target(s) of KPC-1 that are important for PVD dendritic arborization.

## Materials and Methods

### Strains and genetics

Worms were grown on OP50 *Escherichia coli*-seeded nematode growth medium plates at 20°C. Strains used in this work include: N2 (wild type reference), *kpc-1(dz177)*, *kpc-1(dz182)*, *kpc-1(dz185)*, *kpc-1(gk333538)*, *kpc-1(gk779937)*, *kpc-1(gk8)*, *sax-7(nj48)*, *sax-7(dz156)*, *mnr-1(dz175)* and *dma-1(tm5159)*. PVD neurons were visualized by the integrated transgene *wdIs52 (Is[F49H12.4::GFP])* and FLP neurons with *muIs32 (Is[mec-7^prom^::GFP])*. Transgenic strains for cell-specific rescue were established by injecting the respective plasmids at 5 ng/µl together with *rol-6 (su1006)* at 50 ng/µl as a dominant injection marker into *kpc-1(dz182)*. The transcriptional reporter *kpc-1^prom5.8^::GFP* was injected at 5 ng/µl together with *ttx-3^prom^::mCherry* at 5 ng/µl into N2 wild type animals. For details on strains and transgenesis see also Supplementary Text S1.

### Identification of *kpc-1*

In a clonal F1 Ethyl methanesulfonate (EMS) screen [16] we identified three alleles of *kpc-1*. Two alleles with similar phenotypes, *dz177* and *dz182* were mapped and cloned using a one-step whole genome sequencing approach [28]. Within the mapped region both *dz177* and *dz182* carried nonsense mutations in *kpc-1* on chromosome I at positions 11,676,957 (C to T) and 11,679,245 (G to A) (WS220), respectively. One additional allele, *dz185* failed to complement *dz182* for the PVD phenotype and contained a missense mutation in *kpc-1* at position 11,678,076 (A to T); three additional alleles were obtained from the *C. elegans* strain collection: the *gk8* deletion allele and the missense alleles *gk333538* and *gk779937*, which change 11,678,078 (G to A) and 11,678,071 (G to A), respectively. Transgenic animals carrying a wild type copy of the *kpc-1* locus (fosmid WRM0635bG07) fully rescued the PVD defect in *dz182* mutants (Figure 2C). For details on mapping and identifying the molecular lesions of different alleles see Supplementary Text S1, and Tables S1 & S2.

### Molecular cloning

The *kpc-1* cDNA was amplified with gene specific primers from a N2 mixed stage cDNA sample and cloned *Kpn*I/*Eco*RI downstream of the *ttx-3^promB^* regulatory element [29]. For the cell specific heterologous rescue the *kpc-1* cDNA was placed under control of the *dpy-7^prom^*, *myo-3^prom^*, or *ser-2^prom3^* promoters, respectively. The transcriptional reporter was constructed by cloning 5.8 kb upstream of the predicted *kpc-1* translational start site into *pPD95.75* (gift of A. Fire).

### Imaging and quantification of branching

Synchronized starved L1 larvae were allowed to grow for 30 hrs (corresponding to mid- to late L4) at which time they were mounted and fluorescent images of immobilized animals (1–5 mM levamisol, Sigma) were captured using a Zeiss Axioimager Z1 Apotome. *Z* stacks were collected and maximum projections were used for tracing of dendrites as described [16]. For time lapse imaging, animals at the L3 stage (by gonadal development) were immobilized as described [30]. PVD neurons were imaged for six to eight hours in 5 min intervals starting at the beginning of secondary branch development. Z-projections (0.5 µm/step) spanning the focal depth of the neuron were collected using a 63× objective. At least four movies per genotype were obtained using an inverted Nikon TE2000-S microscope equipped with a Perkin-Elmer UltraVIEW spinning disk unit. Volocity software (version 6.2.1) was used to collect the raw files. Processing was carried out using the Image-J 1.46r software.

## Supporting Information

Figure S1 Multiple sequence alignment of KPC-1 splice variants with human and yeast enzymes. Multiple sequence alignment of the KPC-1 furin-like proprotein convertase with human proprotein convertases as indicated and the yeast Kex2 subtilisin-like protease using Multalin (http://multalin.toulouse.inra.fr/multalin/). The alignment was rendered using Boxshade (http://www.ch.embnet.org/software/BOX_form.html). Allelic changes are shown. The deletion allele *gk8* deletes exons 3, 4 and part of exon 5, resulting in a frameshift after L185 and a predicted stop-codon after 17 non-homologous residues following the Leucine residue in both isoforms. Accession numbers: KPC-1A: NP_492974, KPC-1B: NP_001021101, furin: NP_002560, PCSK4: NP_060043, PCSK5: NP_006191, PCSK6: NP_002561, and Kex2p: NP_014161. *Ce*: *Caenorhabditis elegans*, *Hs*: *Homo sapiens*, *Sc*: *Saccharomyces cerevisiae*.
(TIF)

Figure S2 PVD defects occur early during PVD development. **A–C** Epifluorescent micrograph of PVD in animals at different developmental stages (L3 & L4 larval stage and adult). Scale bars indicate 20 µm.
(TIF)

Figure S3 Overexpression of KPC-1 in wild type animals does not result in defects in PVD dendrite arborization. Quantification of secondary branch numbers/100 µm anterior to the PVD cell body in wild type, *kpc-1(gk8)*, and animals that cell specifically overexpress the *kpc-1* cDNA under the *ser-2^prom3^* promoter (*PVD::kpc-1*). Data are represented as mean +/− SEM. Statistical comparisons were performed using one-sided ANOVA with the Tukey correction and statistical significance is indicated (****, *P*≤0.0001, ns: not significant (*P*>0.05)).
(TIF)

Figure S4 Expression of the *kpc-1^prom2.8^::GFP and kpc-1^prom5.8^::GFP* reporters. **A** Schematic of the *kpc-1* locus on linkage group (LG) I. The transcriptional *kpc-1::GFP* reporter that contains 2.8 kb of regulatory sequence upstream of the ATG (*kpc-1^prom2.8^::GFP*) is shown. **B** Composite epifluorescent micrograph of an adult transgenic animal carrying the *kpc-1^prom2.8^::GFP* transgene. Expression is seen in the pharynx (ph) and the nervous system, *e.g.* the phasmids, ventral cord motor neurons (mn) but not in PVD mechanosensory neurons. Whereas widespread expression in the nervous system is seen, no expression in PVD is observed in the right lateral posterior section of the worm which is indicated by a white arrowhead. **C–F** Expression of the *kpc-1^prom5.8^::GFP* reporter at different developmental stages as indicated (emb: embryo). C′ is a brightfield image of C. Scale bars indicate 10 µm for the embryo and 100 µm for all other images. Cells, putatively identified as PVD, are circled in orange.
(TIF)

Figure S5 Quantification of aggregate branch length in *kpc-1* mutant animals. **A**–**C** Quantification of aggregate branch length of secondary, tertiary, and quaternary per 100 µm anterior to the PVD cell body. Data are represented as mean +/− SEM. Statistical comparisons were performed using one-sided ANOVA with the Tukey correction and statistical significance is indicated (*, *P*≤0.05; **, *P*≤0.01; ***, *P*≤0.001; ****, *P*≤0.0001, ns: not significant (*P*>0.05)). N = 21 animals (1371 dendritic branches) of wild type control; N = 21 animals (1158 dendritic branches) of *kpc-1(gk8)*; N = 21 animals (1154 dendritic branches) of *mnr-1(dz175)*; N = 22 animals (1251 dendritic branches) of *sax-7(nj48)*, N = 22 animals (520 dendritic branches) of *dma-1(tm5159)*, N = 19 animals (1119 dendritic branches) of *kpc-1(gk8)*; *mnr-1(dz175*), N = 25 animals (1213 dendritic branches) of *kpc-1(gk8)*; *sax-7(nj48)*, N = 22 animals (319 dendritic branches) of *kpc-1(gk8)*; *dma-1(tm5159)*. **D** Quantification of total aggregate length of all branches per 100 µm anterior to the PVD cell body. Data are represented as mean +/− SEM. Statistical comparisons were performed using one-sided ANOVA with the Tukey correction. Statistical significance is indicated (****, *P*<0.0005). Number of animals and dendrites scored as in Figure S5A–C.
(TIF)

Figure S6 The defects in *kpc-1* mutant animals are specific and not caused by defects in neuropeptide processing. **A**–**C** Images of adult animals carrying mutations in other proprotein convertases. For a control image of animals raised at 20°C see Fig. 1A. Anterior is to the right in panels A–G and ventral down (except for C) and arrowheads indicate the PVD axon. Scale bar: 20 µm. **D**–**G** Images of adult animals carrying mutations in genes required for neuropeptide processing (E,F) or secretion (G). D is a control image of animal raised at 25°C to compare with the temperature-sensitive mutation *egl-21(n611)* 25°C, the non-permissive temperature. **H** Locus and gene model of the *aex-5/kpc-3* on chromosome I. Indicated is the location of the *gk419962* nonsense allele, which results in a premature stop codon after 286 amino acids. The resulting truncated protein lacks 119 amino acids of the conserved 291 protease domain and is thus likely a strong if not complete loss of function allele. **I** Locus and gene model of the *pamn-1* on chromosome I which encodes the peptidyl-α-hydroxyglycine-α-amidating lyase. Indicated is the extent of the *ok2681* allele which deletes 519 nucleotides. The predicted mRNA encodes a protein with a frameshift after 140 amino acids resulting in a premature stop codon after 4 non-homologous amino acids. This allele is a strong if not complete loss of function allele.
(TIF)

Table S1 Whole genome sequencing statistics. Shown are whole genome sequencing statistics of the *kpc-1(dz177)* and *kpc-1(dz182)* alleles as indicated.
(DOCX)

Table S2 Polymorphisms identified by whole genome sequencing within the mapped region. List of all polymorphisms found in *kpc-1(dz177)* and *kpc-1(dz182)* alleles within the mapped reagion.
(DOCX)

Table S3 List of predicted furin cleavage sites in candidate proteins. List of proteins known to be involved in PVD development with sites predicted to be cleaved by proprotein convertases such as furin.
(TIF)

Movie S1 PVD development in a wild type animal. Lateral view of the left PVD, showing the establishment of secondary, tertiary (ventral side, bottom) and quaternary branches (dorsal side, top) in an animal at the L3 larval stage. The PVD cell body is the biggest brighter spot on the right. Small bright spots are part of the gut auto-fluorescence. The PVD neuron was visualized with GFP in the *wdIs52* transgenic strain [36].
(MOV)

Movie S2 PVD development in a *kpc-1(gk8)* mutant animal. Ectopic tertiary PVD branching in *kpc-1(gk8)* null mutant animal at the L3 larval stage. Shown is the PVD neuron sprouting short secondary branches with frequent ectopic short tertiary branches. The PVD neuron was visualized in the *kpc-1(gk8); wdIs52* strain.
(MOV)

Text S1 The Supplementary Text provides details Materials and Methods, specifically on the strains used as well as on the cloning of the mutations identified in the genetic screen.
(DOCX)
