# Supplementary figures and images for: The Proprotein Convertase KPC-1/Furin Controls Branching and Self-avoidance of Sensory Dendrites in Caenorhabditis elegans
Source: PLoS Genet. 2014 Sep 18;10(9):e1004657. doi: 10.1371/journal.pgen.1004657 (PMC4169376; doi:10.1371/journal.pgen.1004657)

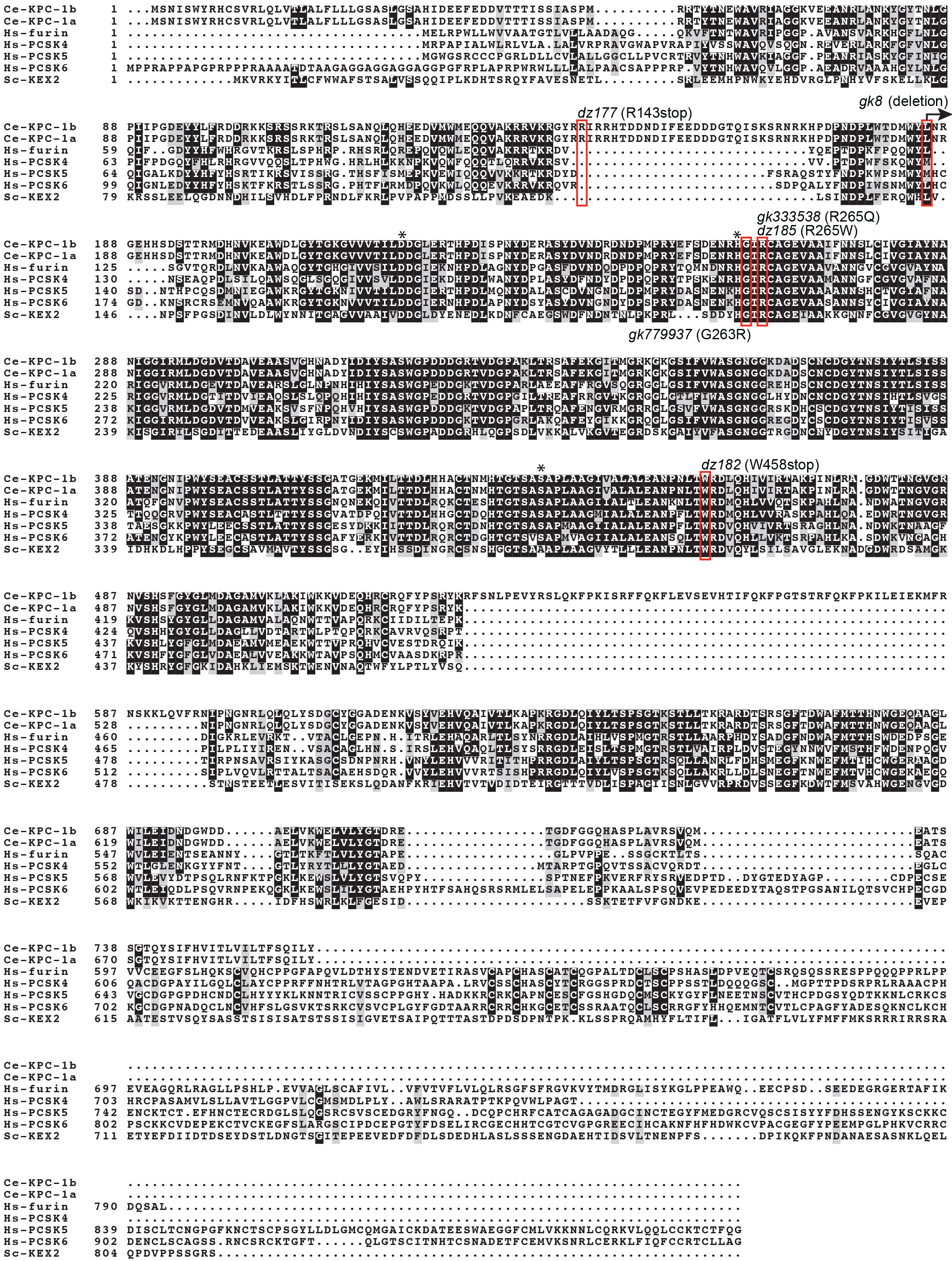

Supplement: Figure S1 — Multiple sequence alignment of KPC-1 splice variants with human and yeast enzymes. Multiple sequence alignment of the KPC-1 furin-like proprotein convertase with human proprotein convertases as indicated and the yeast Kex2 subtilisin-like protease using Multalin (http://multalin.toulouse.inra.fr/multalin/). The alignment was rendered using Boxshade (http://www.ch.embnet.org/software/BOX_form.html). Allelic changes are shown. The deletion allele gk8 deletes exons 3, 4 and part of exon 5, resulting in a frameshift after L185 and a predicted stop-codon after 17 non-homologous residues following the Leucine residue in both isoforms. Accession numbers: KPC-1A: NP_492974, KPC-1B: NP_001021101, furin: NP_002560, PCSK4: NP_060043, PCSK5: NP_006191, PCSK6: NP_002561, and Kex2p: NP_014161. Ce: Caenorhabditis elegans, Hs: Homo sapiens, Sc: Saccharomyces cerevisiae. (TIF) [file pgen.1004657.s001.tif]

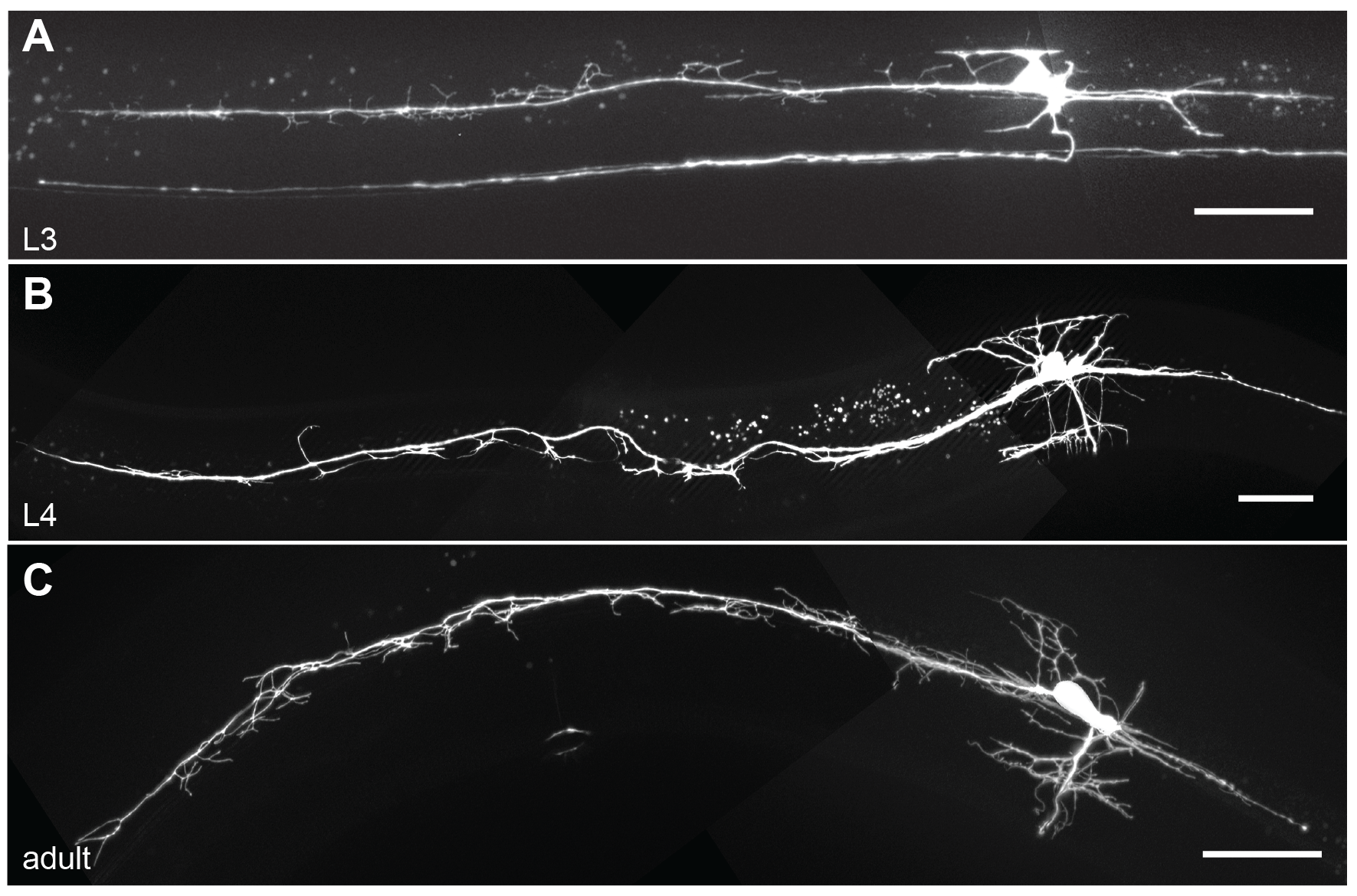

Supplement: Figure S2 — PVD defects occur early during PVD development. A–C Epifluorescent micrograph of PVD in animals at different developmental stages (L3 & L4 larval stage and adult). Scale bars indicate 20 µm. (TIF) [file pgen.1004657.s002.tif]

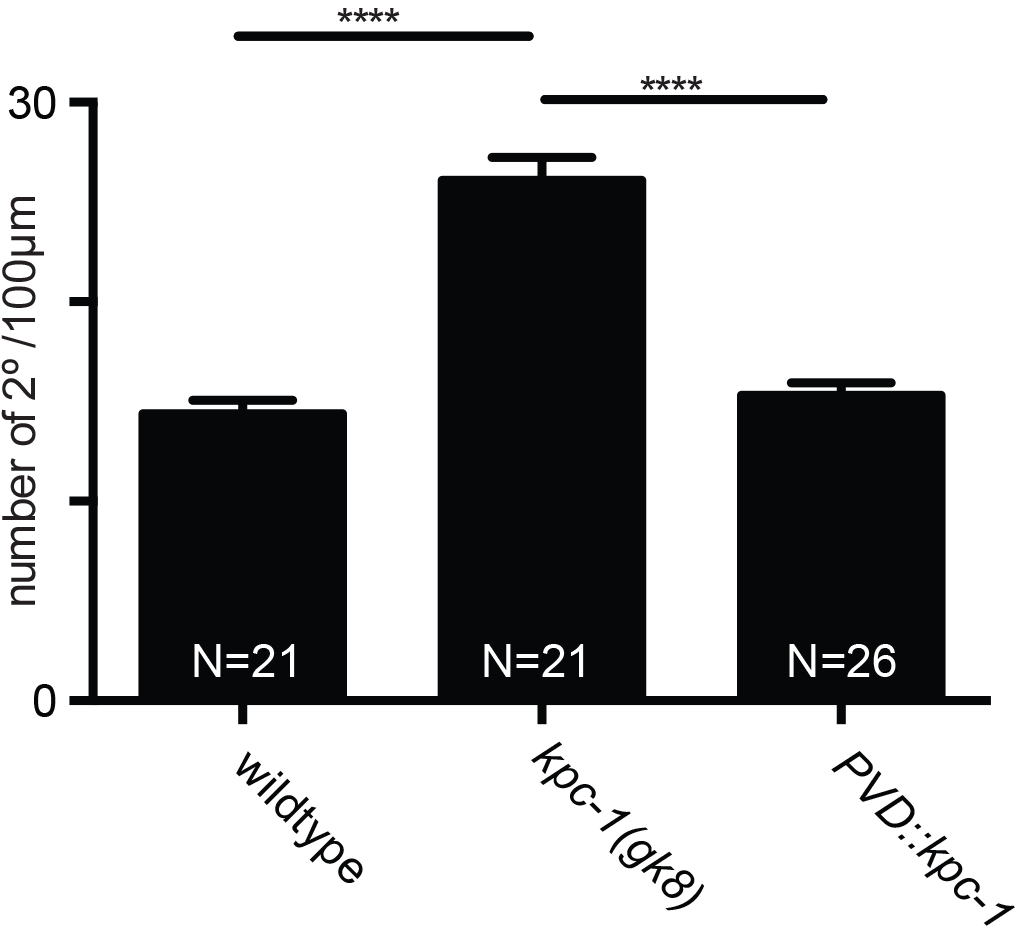

Supplement: Figure S3 — Overexpression of KPC-1 in wild type animals does not result in defects in PVD dendrite arborization. Quantification of secondary branch numbers/100 µm anterior to the PVD cell body in wild type, kpc-1(gk8), and animals that cell specifically overexpress the kpc-1 cDNA under the ser-2prom3 promoter (PVD::kpc-1). Data are represented as mean +/− SEM. Statistical comparisons were performed using one-sided ANOVA with the Tukey correction and statistical significance is indicated (****, P≤0.0001, ns: not significant (P>0.05)). (TIF) [file pgen.1004657.s003.tif]

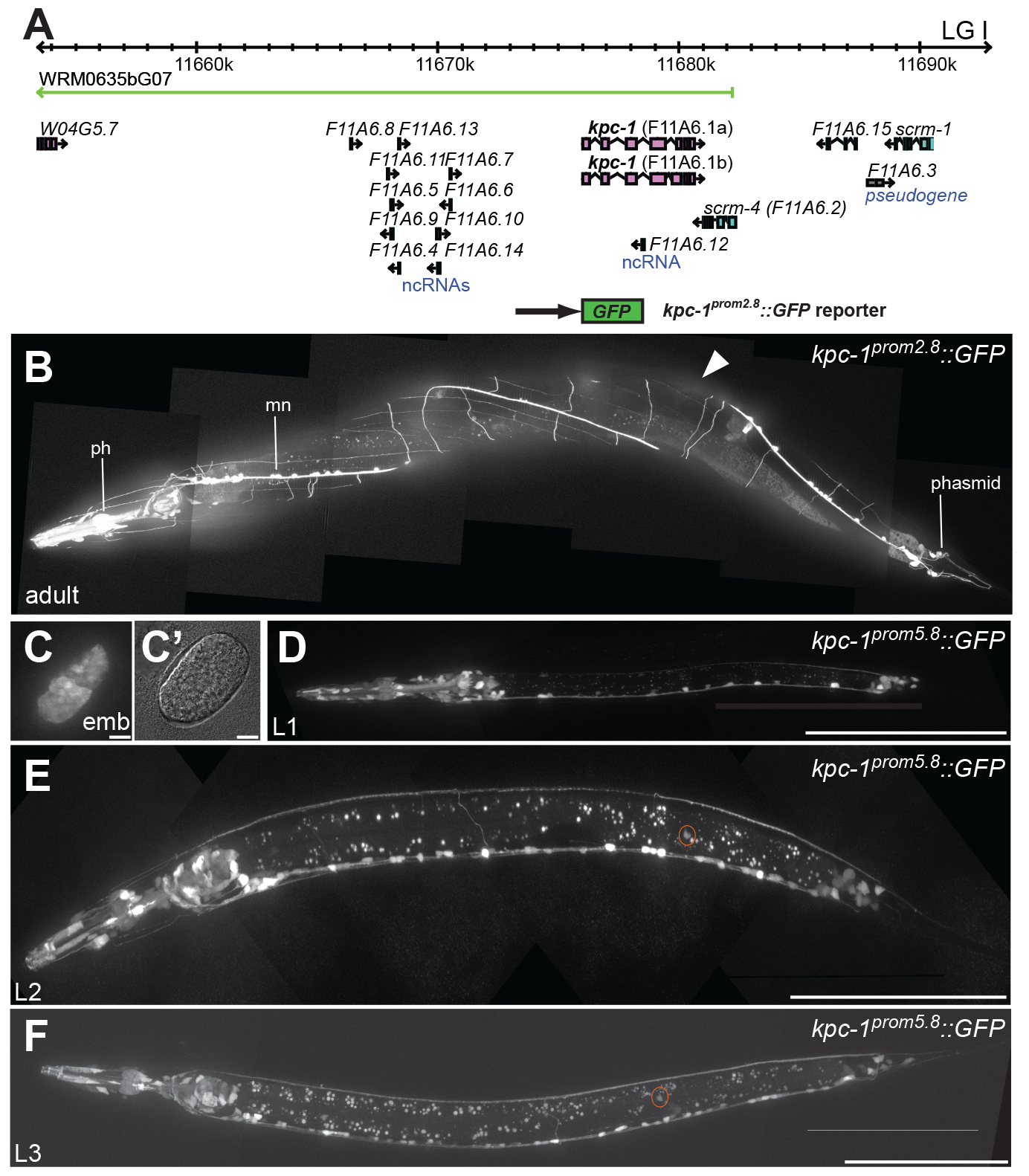

Supplement: Figure S4 — Expression of the kpc-1prom2.8::GFP and kpc-1prom5.8::GFP reporters. A Schematic of the kpc-1 locus on linkage group (LG) I. The transcriptional kpc-1::GFP reporter that contains 2.8 kb of regulatory sequence upstream of the ATG (kpc-1prom2.8::GFP) is shown. B Composite epifluorescent micrograph of an adult transgenic animal carrying the kpc-1prom2.8::GFP transgene. Expression is seen in the pharynx (ph) and the nervous system, e.g. the phasmids, ventral cord motor neurons (mn) but not in PVD mechanosensory neurons. Whereas widespread expression in the nervous system is seen, no expression in PVD is observed in the right lateral posterior section of the worm which is indicated by a white arrowhead. C–F Expression of the kpc-1prom5.8::GFP reporter at different developmental stages as indicated (emb: embryo). C′ is a brightfield image of C. Scale bars indicate 10 µm for the embryo and 100 µm for all other images. Cells, putatively identified as PVD, are circled in orange. (TIF) [file pgen.1004657.s004.tif]

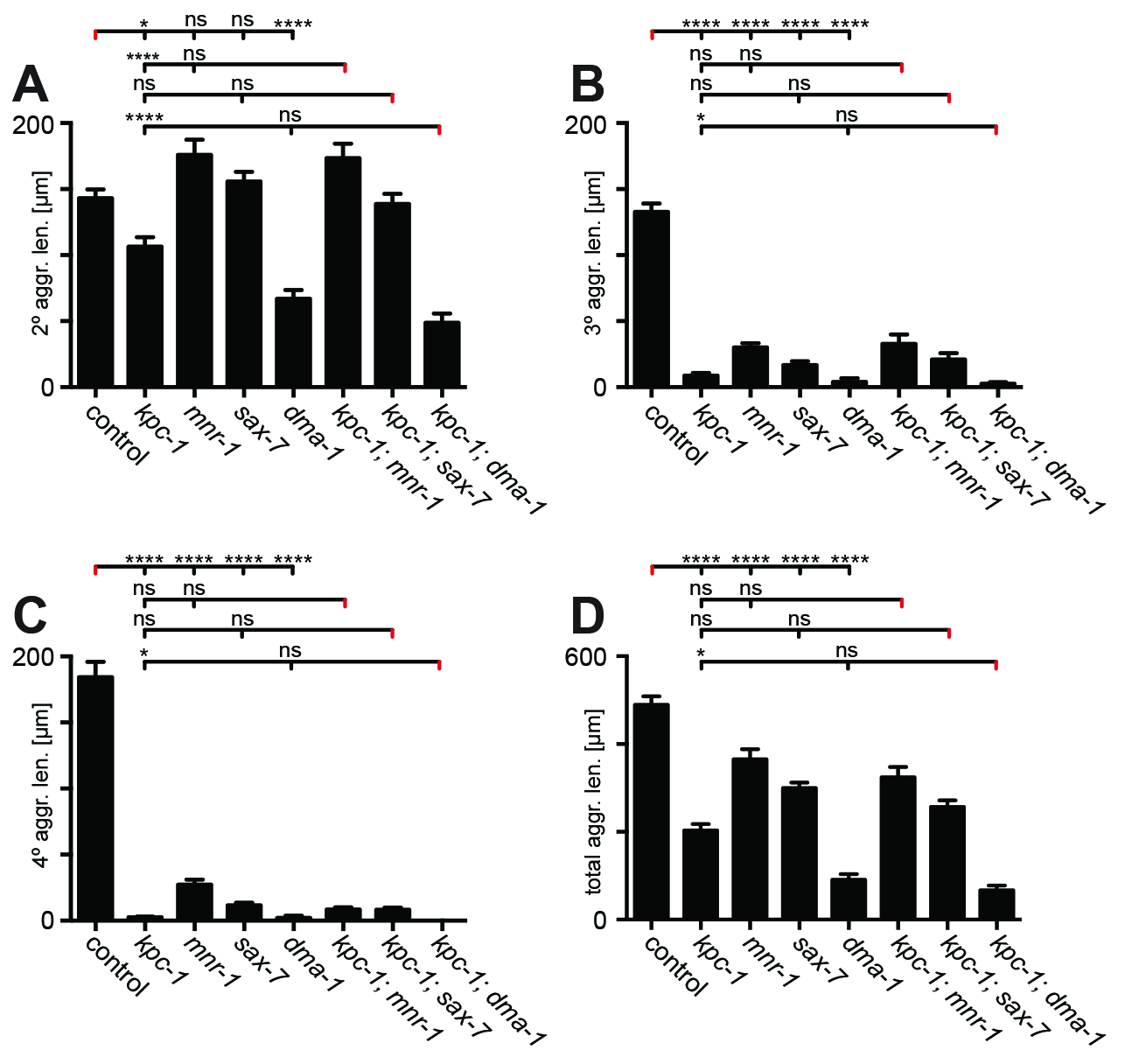

Supplement: Figure S5 — Quantification of aggregate branch length in kpc-1 mutant animals. A–C Quantification of aggregate branch length of secondary, tertiary, and quaternary per 100 µm anterior to the PVD cell body. Data are represented as mean +/− SEM. Statistical comparisons were performed using one-sided ANOVA with the Tukey correction and statistical significance is indicated (*, P≤0.05; **, P≤0.01; ***, P≤0.001; ****, P≤0.0001, ns: not significant (P>0.05)). N = 21 animals (1371 dendritic branches) of wild type control; N = 21 animals (1158 dendritic branches) of kpc-1(gk8); N = 21 animals (1154 dendritic branches) of mnr-1(dz175); N = 22 animals (1251 dendritic branches) of sax-7(nj48), N = 22 animals (520 dendritic branches) of dma-1(tm5159), N = 19 animals (1119 dendritic branches) of kpc-1(gk8); mnr-1(dz175), N = 25 animals (1213 dendritic branches) of kpc-1(gk8); sax-7(nj48), N = 22 animals (319 dendritic branches) of kpc-1(gk8); dma-1(tm5159). D Quantification of total aggregate length of all branches per 100 µm anterior to the PVD cell body. Data are represented as mean +/− SEM. Statistical comparisons were performed using one-sided ANOVA with the Tukey correction. Statistical significance is indicated (****, P<0.0005). Number of animals and dendrites scored as in Figure S5A–C. (TIF) [file pgen.1004657.s005.tif]

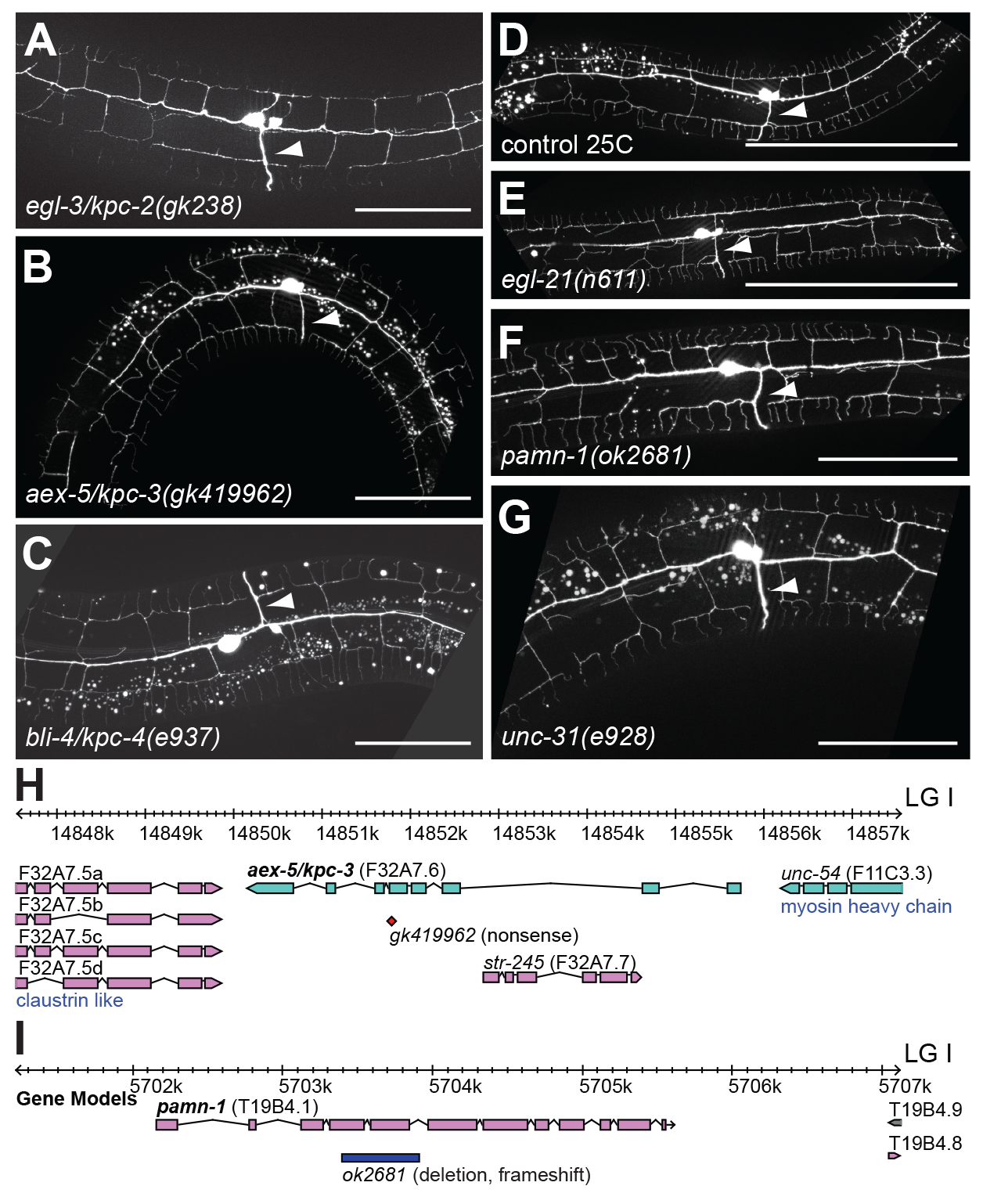

Supplement: Figure S6 — The defects in kpc-1 mutant animals are specific and not caused by defects in neuropeptide processing. A–C Images of adult animals carrying mutations in other proprotein convertases. For a control image of animals raised at 20°C see Fig. 1A. Anterior is to the right in panels A–G and ventral down (except for C) and arrowheads indicate the PVD axon. Scale bar: 20 µm. D–G Images of adult animals carrying mutations in genes required for neuropeptide processing (E,F) or secretion (G). D is a control image of animal raised at 25°C to compare with the temperature-sensitive mutation egl-21(n611) 25°C, the non-permissive temperature. H Locus and gene model of the aex-5/kpc-3 on chromosome I. Indicated is the location of the gk419962 nonsense allele, which results in a premature stop codon after 286 amino acids. The resulting truncated protein lacks 119 amino acids of the conserved 291 protease domain and is thus likely a strong if not complete loss of function allele. I Locus and gene model of the pamn-1 on chromosome I which encodes the peptidyl-α-hydroxyglycine-α-amidating lyase. Indicated is the extent of the ok2681 allele which deletes 519 nucleotides. The predicted mRNA encodes a protein with a frameshift after 140 amino acids resulting in a premature stop codon after 4 non-homologous amino acids. This allele is a strong if not complete loss of function allele. (TIF) [file pgen.1004657.s006.tif]

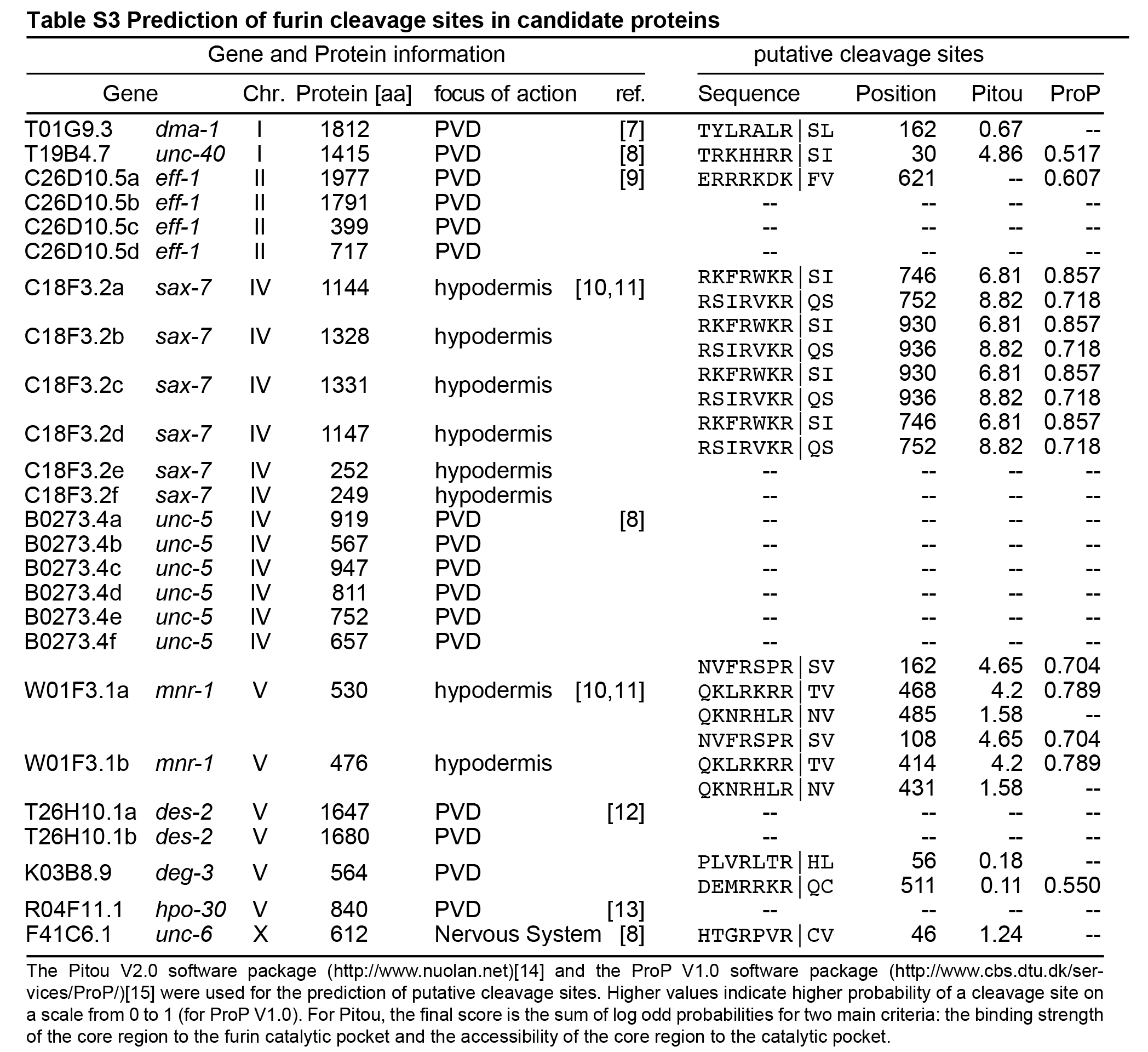

Supplement: Table S3 — List of predicted furin cleavage sites in candidate proteins. List of proteins known to be involved in PVD development with sites predicted to be cleaved by proprotein convertases such as furin. (TIF) [file pgen.1004657.s009.tif]
