# Supplementary material for: The Proprotein Convertase KPC-1/Furin Controls Branching and Self-avoidance of Sensory Dendrites in Caenorhabditis elegans
Source: PLoS Genet. 2014 Sep 18;10(9):e1004657. doi: 10.1371/journal.pgen.1004657 (PMC4169376; doi:10.1371/journal.pgen.1004657)
Supplement: Table S1 — Whole genome sequencing statistics. Shown are whole genome sequencing statistics of the kpc-1(dz177) and kpc-1(dz182) alleles as indicated. (DOCX) [file pgen.1004657.s007.docx]

**Table S1. Whole genome sequencing statistics**

|  | ***dz177*** | ***dz182*** |
| --- | --- | --- |
| **# N2/HI recombinants pooled** | 30 | 41 |
| **Average read depth** | 33x | 39x |
| **# splice site mutations ^a^** | 3 | 6 |
| **# synonymous mutations** | 87 | 79 |
| **# missense mutations** | 85 | 76 |
| **# nonsense mutations** | 2 | 3 |

^a^ mutations refer to homozygous variants not shared by both strains as compared to the *C. elegans* reference genome (WB220).
