# Supplementary material for: The Proprotein Convertase KPC-1/Furin Controls Branching and Self-avoidance of Sensory Dendrites in Caenorhabditis elegans
Source: PLoS Genet. 2014 Sep 18;10(9):e1004657. doi: 10.1371/journal.pgen.1004657 (PMC4169376; doi:10.1371/journal.pgen.1004657)
Supplement: Table S2 — Polymorphisms identified by whole genome sequencing within the mapped region. List of all polymorphisms found in kpc-1(dz177) and kpc-1(dz182) alleles within the mapped reagion. (DOCX) [file pgen.1004657.s008.docx]

**Table S2. Polymorphisms identified by whole genome sequencing within the mapped region**

| **LG** | **Position^a^** | | **Ref** | **Change** | **Quality** | **Coverage** | **Gene ID** | **Gene name** | **Effect^b^** | **Old AA/**  **newAA** |
| --- | --- | --- | --- | --- | --- | --- | --- | --- | --- | --- |
| ***dz177*** | |  |  |  |  |  |  |  |  |  |
| I^c^ | 9246867 | | G | C | 639.8 | 26 | F36H2.2 | *F36H2.2* | NS^a^ | R/G |
| I | 9252694 | | C | T | 1324.96 | 42 | F36H2.5 | *F36H2.5* | stop | W/* |
| I | 9699161 | | T | C | 1006.46 | 35 | C04F12.8 | *C04F12.8* | NS | N/S |
| I | 9955459 | | C | T | 487.46 | 24 | T24D1.2 | *T24D1.2* | NS | E/K |
| I | 10269199 | | T | A | 48.88 | 15 | ZC247.1 | *ZC247.1* | NS | M/L |
| I | 10269541 | | A | T | 35.63 | 4 | ZC247.1 | *ZC247.1* | NS | S/T |
| I | 10390849 | | C | T | 969.62 | 31 | F45H11.4 | *mgl-2* | SA |  |
| I | 11144034 | | C | T | 939.88 | 32 | ZK39.4 | *clec-93* | NS | P/L |
| I | 11355256 | | C | T | 242.45 | 11 | F56G4.2 | *pes-2.1* | NS | S/F |
| **I** | **11676957** | | **C** | **T** | **568.08** | **24** | **F11A6.1** | *kpc-1* | **stop** | **R/*** |
| ***dz182*** | |  |  |  |  |  |  |  |  |  |
| I | 4400309 | | G | A | 708.16 | 28 | *T21E12.4* | *dhc-1* | NS | A/T |
| I | 4670084 | | G | A | 785.22 | 26 | *B0041.7* | *xnp-1* | NS | L/F |
| I | 5042625 | | * |  | 753.36 | 20 | *C46H11.7* | *C46H11.7* | del | T/- |
| I | 5432781 | | G | A | 391.69 | 15 | *F27C1.8* | *dpy-5* | Stop | Q/* |
| I | 5891041 | | A | T | 1172.38 | 39 | *C34G6.4* | *pgp-2* | NS | E/D |
| I | 5998603 | | G | A | 1107.74 | 36 | *C06A5.7* | *unc-94* | NS | L/F |
| I | 6066250 | | * |  | 4868.96 | 21 | *C27A12.9* | *C27A12.9* | Stop | -/F*L*LEI |
| I | 6438603 | | G | A | 428.12 | 16 | *T23H2.2* | *snt-4* | NS | G/D |
| I | 6526827 | | T | A | 621.35 | 21 | *T10E9.4* | *T10E9.4* | NS | L/M |
| I | 6555655 | | G | A | 662.7 | 25 | *F57B10.7* | *tre-1* | NS | G/E |
| I | 6562172 | | G | A | 959.98 | 33 | *F57B10.6* | *xpg-1* | NS | A/T |
| I | 6801829 | | * |  | 1586.34 | 32 | *E02D9.1* | *E02D9.1* | SA | |
| I | 6841291 | | C | A | 591.8 | 25 | *K02F2.1* | *dpf-3* | NS | T/K |
| I | 6934829 | | G | A | 724.54 | 24 | *T10B11.2* | *T10B11.2* | NS | A/T |
| I | 7301404 | | C | G | 705.96 | 23 | *F13G3.5* | *ttx-7* | NS | G/A |
| I | 7607997 | | G | A | 708.47 | 25 | *D2030.10* | *aex-1* | NS | G/R |
| I | 7638240 | | G | A | 951.61 | 30 | *F29D11.2* | *capg-1* | NS | L/F |
| I | 8293326 | | G | A | 1107.71 | 38 | *T01G9.4* | *npp-2* | NS | R/C |
| I | 8367094 | | G | A | 1045.47 | 32 | *F16D3.4* | *F16D3.4* | NS | S/L |
| I | 9025493 | | G | A | 512.42 | 19 | *F36F2.5* | *tax-2* | NS | D/N |
| I | 9246867 | | G | C | 559.12 | 19 | *F36H2.2* | *F36H2.2* | NS | R/G |
| I | 9664604 | | G | A | 766.48 | 25 | *C03D6.4* | *npp-14* | NS | T/M |
| I | 9699161 | | T | C | 681.01 | 22 | *C04F12.8* | *C04F12.8* | NS | N/S |
| I | 9713696 | | G | A | 663.82 | 26 | *C16C2.4* | *C16C2.4* | NS | P/S |
| I | 9907219 | | G | A | 457.39 | 22 | *F52F12.6* | *ztf-11* | NS | T/I |
| I | 10113280 | | * |  | 808.26 | 24 | *Y106G6D.3* | *Y106G6D.3* | fs | |
| I | 11145688 | | * |  | 856.25 | 20 | *ZK39.9* | *ZK39.9* | fs | |
| **I** | **11679245** | | **G** | **A** | **372.15** | **17** | ***F11A6.1*** | ***kpc-1*** | **stop** | **W/*** |

^a^ For *dz177* polymorphisms between 9,000,000 and 12,000,000 and for *dz182* between 4,000,000 and 12,000,000 in linkage group II were considered (see Fig. S1).

^b^ NS: non-synonymous change, stop: stop codon gained, SA: splice acceptor mutation, del: deletion, fs: frame shift.

^c^ Polymorphisms shaded in gray are identical between both sets and polymorphisms shaded in red are affecting the same gene, but in different positions (*kpc-1*). Shaded in green is a nonsense mutation in *dpy-5.*
