# Supplementary material for: The Proprotein Convertase KPC-1/Furin Controls Branching and Self-avoidance of Sensory Dendrites in Caenorhabditis elegans
Source: PLoS Genet. 2014 Sep 18;10(9):e1004657. doi: 10.1371/journal.pgen.1004657 (PMC4169376; doi:10.1371/journal.pgen.1004657)
Supplement: Text S1 — The Supplementary Text provides details Materials and Methods, specifically on the strains used as well as on the cloning of the mutations identified in the genetic screen. (DOCX) [file pgen.1004657.s012.docx]

**SUPPLEMENTARY TEXT S1**

**Supplementary Materials and Methods**

***Strain list***

*Fluorescent reporter strains*

PVD:

*otIs138* [*ser-2 ^prom3^*::GFP]X [[9](#_ENREF_9)].

*wdIs52* [*F49H12.4 ^prom^*::GFP]II [[36](#_ENREF_36)].

FLP:

*muIs32* [*mec-7 ^prom^*::GFP]II [[37](#_ENREF_37)].

VC Neurons:

*vsIs13* [*pes-10^prom^* ::GFP] IV [[38](#_ENREF_38)]

AIY:

*otIs76mgIs18* [*ttx-3^prom^ ::kal-1; ttx-3^prom^* ::GFP] IV [[23](#_ENREF_23)]

DD, VD and AVL neurons:

*juIs76* [*unc-25^prom^* ::GFP] IV [[39](#_ENREF_39)]

D type motor neurons:

*evIs82b* [*unc-129^prom^* ::GFP] IV [[40](#_ENREF_40)]

Touch receptor neurons:

*muIs32* [*mec-7^prom^* ::GFP] II [[37](#_ENREF_37)]

HSN:

*zdIs13* [*tph-1^prom^* ::GFP] IV [[41](#_ENREF_41)]

*Mutant strains*

EB1467: *kpc-1(dz177)I;wdIs52,* 5x backcrossed

EB1469: *kpc-1(dz182)I;wdIs52,* 6x backcrossed

EB1313: *kpc-1(dz185)I;wdIs52*

EB1954: *kpc-1(gk333538)I;wdIs52*

KR2838: *hDf17/hIn1[unc-54(h1040)] I*

VC461: *egl-3(gk238) V*

CB937: *bli-4(e937) I*

VC30124: *aex-5(gk419962)I*

EB1271: *mnr-1(dz175)V;wdIs52*

EB1564: *dma-1(tm5159)I;wdIs52*

EB1753: *sax-7(nj48)IV;wdIs52*

EB1908: *kpc-1(gk8)I; mnr-1(dz175)V; wdIs52*

EB1752: *kpc-1(gk8)I; sax-7(nj48)IV; wdIs52*

EB1906: *dma-1(tm5159)I; kpc-1(gk8)I; wdIs52*

EB1649: *dzIs43[myo-3 prom::mnr-1]; wdIs52*

EB2377: *kpc-1(gk8)I; dzIs43[myo-3 prom::mnr-1]; wdIs52*

EB2378: *kpc-1(gk8)I; mnr-1(dz175)V; dzIs43[myo-3 prom::mnr-1]; wdIs52*

EB2025: *kpc-1(gk8)I;evIs82b IV*

EB2446: *kpc-1(gk8)I;muIs32 II*

EB2447: *kpc-1(gk8)I;vsIs13 IV*

EB2448: *kpc-1(gk8)I;otIs76mgIs18 IV*

EB2449: *kpc-1(gk8)I;juIs76 IV*

EB2450: *kpc-1(gk8)I;zdIs13 IV*

*Transgenic strains*

*Transcriptional kpc-1 reporters:*

Plasmids were injected at 5 ng/µl together with *ttx-3^prom^::mCherry* at 5 ng/µl and pBluescript to a final concentration of 100 ng/µl into N2 wild type animals

*EB2256: dzEx1259 [kpc-1^prom5.8^::GFP]*

*Fosmid rescue*

Fosmid was injected at 2 ng/µl each together with *myo-3 ^prom^::mCherry* at 50 ng/µl and pBluescript to a final concentration of 100 ng/µl.

EB1469: *kpc-1(dz182)I;* *wdIs52; dzEx667* [fosmid *WRM635bG07*; *myo-3 ^prom^::mCherry*]

*Heterologous rescue of kpc-1 mutants*

Plasmids were injected at 5 ng/µl together with *pRF4* *[rol-6(su1006)]* at 50 ng/µl and pBluescript to a final concentration of 100ng/µl.

EB1567-8 *kpc-1(dz182)I* *wdIs52; dzEx743-4* *[myo-3 ^prom^::kpc-1 pRF4]*

EB1583-5 *kpc-1(dz182)I wdIs52; dzEx747-9 [dpy-7^prom^::kpc-1; pRF4]*

EB1650-1 *kpc-1(dz182)I; wdIs52; dzEx797-8 [ser2prom3::kpc-1; pRF4]*

***Molecular cloning of mutations.***

To identify the molecular lesions in *dz177* and *dz182* we employed a whole genome sequencing approach that combines mapping and sequencing as described [[28](#_ENREF_28)]. Mutant alleles (which were isolated in a N2 Bristol background) were crossed with the polymorphic Hawaiian strain CB4856 and the DNA of 30 (*dz177*) and 41 (*dz182*) homozygous mutant F2s, respectively, was pooled and sequenced using paired end 100bp reads on an Illumina HiSeq2000 (Table S1). Sequencing reads were mapped to the *C. elegans* reference genome (WB220) using CloudMap as described [[28](#_ENREF_28)] (data not shown). Briefly, the ratio of the number of reads showing the Hawaiian SNP divided by the total number of sequence reads for a given position was plotted along the chromosomes. A local regression (LOESS) line was then plotted for each chromosome with default parameters as described [[28](#_ENREF_28)]. The results of SNP mapping identified the same region on the right arm of LG I in both mutants. Within the identified region *dz177* and *dz182* encoded premature stop codons at different positions in the same gene, *F11A6.1* (*kpc-1*), suggesting these nonsense mutations are causative for the phenotype in PVD dendrites (Table S2). Using complementation tests followed by Sanger sequencing we identified *dz185* as an additional allele. *kpc-1(dz185)* fails to complement *dz182* and encodes a missense mutations in *kpc-1* (R265W) that changes a perfectly conserved residue in a conserved alpha helix that is required for positioning the histidine of the catalytic triad [[22](#_ENREF_22)]. The allele *gk8* was obtained from the *Caenorhabditis Genetics Center* (CGC) in strain VC48 and contains a 2238 bp deletion that results in a predicted frameshift after 185 amino acids (Fig. S1). To further confirm causality between the mutations in *kpc-1* and the PVD phenotype, we transgenically rescued the *kpc-1* mutant phenotype by injecting a fosmid (WRM0635bG07) carrying the *kpc-1* locus and found that 1/1 transgenic lines fully rescued the PVD defect in *dz182* mutants (data not shown).

**References**

36. Chatzigeorgiou M, Yoo S, Watson JD, Lee WH, Spencer WC, et al. (2010) Specific roles for DEG/ENaC and TRP channels in touch and thermosensation in C. elegans nociceptors. Nat Neurosci 13: 861-868.

37. Ch'ng Q, Williams L, Lie YS, Sym M, Whangbo J, et al. (2003) Identification of genes that regulate a left-right asymmetric neuronal migration in Caenorhabditis elegans. Genetics 164: 1355-1367.

38. Bany IA, Dong MQ, Koelle MR (2003) Genetic and cellular basis for acetylcholine inhibition of Caenorhabditis elegans egg-laying behavior. J Neurosci 23: 8060-8069.

39. Huang X, Cheng HJ, Tessier-Lavigne M, Jin Y (2002) MAX-1, a novel PH/MyTH4/FERM domain cytoplasmic protein implicated in netrin-mediated axon repulsion. Neuron 34: 563-576.

40. Colavita A, Krishna S, Zheng H, Padgett RW, Culotti JG (1998) Pioneer axon guidance by UNC-129, a C. elegans TGF-beta. Science 281: 706-709.

41. Clark SG, Chiu C (2003) C. elegans ZAG-1, a Zn-finger-homeodomain protein, regulates axonal development and neuronal differentiation. Development 130: 3781-3794.
